# Acculturative Stress among Arab Students in Israel: The Roles of Sense of Coherence and Coping Strategies

**DOI:** 10.3390/ijerph17145106

**Published:** 2020-07-15

**Authors:** Sarah Abu-Kaf, Enas Khalaf

**Affiliations:** Conflict Management and Resolution Program, Department of Multidisciplinary Studies, Ben-Gurion University of the Negev, Beer-Sheva 8410501, Israel; enas.kh88@gmail.com

**Keywords:** acculturative stress, depression, students, Arab, coping strategies, sense of coherence

## Abstract

Background: In Israeli colleges and universities, many Arab students experience acculturative stress. Such stress arises from the need to learn new cultural rules, manage the overarching conflict inherent in maintaining elements of their culture of origin (i.e., Arab culture) while incorporating elements of the host culture (i.e., Jewish academic culture), and deal with experiences of prejudice and discrimination present in the host culture. Methods: This study investigated the association between acculturative stress and depressive symptoms among 170 Arab undergraduates from northern and central Israel. It also explored the roles of sense of coherence and coping strategies in the relationship between acculturative stress and depressive symptoms. Participants completed questionnaires on acculturative stress, depressive symptoms, sense of coherence, coping strategies, and demographics. Results: The findings reveal gender differences in the use of different coping strategies and in levels of depressive symptoms. However, academic-year differences were found only in levels of sense of coherence and depressive symptoms. Specifically, female students expressed higher levels of both active and avoidant coping. Moreover, female students and those in their first and second years of university studies reported higher levels of depressive symptoms. Among the male students, acculturative stress was related to depressive symptoms indirectly via sense of coherence and active coping. In contrast, among the female students, acculturative stress was related to depressive symptoms both directly and indirectly via sense of coherence and avoidant coping. Among first- and second-year students, acculturative stress was related to depressive symptoms indirectly via sense of coherence and avoidant coping. However, among third- and fourth-year students, acculturative stress was related to depressive symptoms both directly and indirectly via sense of coherence. Conclusions: This article underscores the significance of gender and academic-year differences in pathways involving acculturative stress.

## 1. Introduction

### 1.1. Acculturative Stress

Redfield, Linton, and Herskovits [1] defined acculturation as a “phenomenon which results when groups of individuals having different cultures come into continuous first-hand contact with subsequent changes in the original cultural patterns of each other or both groups” (p. 149). The acculturation process can be positive, improving one’s life chances in the new culture. However, it also imposes stress on the individual due to the challenging nature of change and adaptation to new cultural and social expectations. Acculturative stress is usually experienced by those who are in the process of acculturating to the dominant society by adapting the dominant culture’s language and norms [2]. Berry and colleagues [2] described acculturative stress as “a reduction in health status (including psychological, somatic, and social aspects) of individuals who are undergoing acculturation, and for which there is evidence that these health phenomena are related systematically to acculturation phenomena” (p. 491). Drawing on broader stress and adaptation theory (e.g., [3]), Berry [4] claimed that acculturative stress is a stress reaction to life events that are rooted in the experience of acculturation. Individuals experience change events that challenge their cultural understandings about how to live. Such stress arises from multiple aspects of the acculturation process, such as learning new and sometimes confusing cultural rules and expectations, dealing with experiences of prejudice and discrimination, and managing the overarching conflict inherent in maintaining elements of the old culture while incorporating elements of the new [4,5,6]. The aspects of acculturative stress that are salient to college students may relate to language proficiency, unfamiliarity with prevailing cultural practices, cultural self-consciousness, the experience of conflicting value systems, and experiences of discrimination (e.g., [7,8,9]). In previous research, acculturative stress has repeatedly been found to be associated with mental-health problems such as anxiety and depression, feelings of alienation, identity confusion, and heightened levels of psychosomatic symptoms [2,10,11,12]. Among international college students and college students from cultural-ethnic minorities, acculturative stress has been associated with a number of psychological challenges, including depression [13,14].

### 1.2. Protective Factors: Sense of Coherence and Coping Strategies

General models of stress posit that perceiving a situation as being threatening or beyond one’s coping abilities causes stress and leads to negative affect [3,15]. Resilience studies (e.g., [16]) have highlighted the importance of identifying protective factors that reduce the negative effects of stressful events and encourage positive outcomes. Sense of coherence and coping strategies have been identified as the main stress-buffering variables.

#### 1.2.1. Sense of Coherence

Sense of coherence (SOC) refers to a permanent attitude according to which individuals view and understand life and is a measure of the capacity to assess and use available resistance resources to maintain and improve health when faced with stressful situations [17]. In other words, SOC can be defined as a way of viewing life and the ability to manage the stressors that are faced in life [18].

According to Antonovsky [17], SOC is an important resource that enables people to manage stress, to evaluate their external and internal resources, and to identify and use those resources, in order to promote effective coping and adjustment. SOC explains why individuals experiencing stressful or challenging events in their lives are capable of dealing with them [17]. This sense develops during childhood and early adulthood and stabilizes around the age of 30 (in the period of early adulthood [19]). However, Eriksson [20] claimed that SOC tends to increase with age over one’s whole lifespan. Researchers have argued that SOC is a construct that develops differently, according to environmental characteristics and life experiences [21]. SOC integrates three components: comprehensibility, manageability, and meaningfulness. Comprehensibility refers to the individual’s ability to perceive life events as comprehensible and consistent, and to reasonably predict what will happen in the future. Manageability refers to the ability of the individual to understand that the resources at one’s disposal are sufficient to cope with life’s difficulties. Meaningfulness is the extent to which an individual feels that life makes sense emotionally [19]. Meaningfulness motivates an individual to seek resolutions to events or situations that are considered stressful [22]. Antonovsky claimed that an ability to define stressors as irrelevant, neutral, or even as a challenge indicates that a person has a strong SOC, while considering a stressor as endangering one’s well-being is indicative of low SOC [19]. Previous literature has stated that SOC has important positive effects on reactions to stress, as well as problem-solving and emotional coping in general, particularly among individuals who are members of ethnic and cultural minorities [23,24,25,26]. Previous research has found that an individual with a strong SOC is more likely to feel less stress and to have more social support that can be called upon in his or her efforts to cope with stress [19,27]. Previous cross-sectional studies have reported a significant inverse correlation between SOC and depression [28]. Researchers have also found that a strong SOC is associated with fewer depressive symptoms [29]. According to a review conducted by Eriksson and Lindström [30], SOC is strongly, negatively related to perceived depression; stronger SOC is associated with fewer symptoms of perceived depression.

Previous research on SOC in Eastern-collectivistic contexts such as the Arab minority in Israel has revealed two important findings. First, researchers found lower levels of SOC among the Arab minority [23,31]. Second, over time, changes in SOC levels were observed (higher levels) and SOC became a strong predictor of stress reactions [24]. However, this important resource has not been previously examined among the Arab student population and it is important to evaluate the levels and implications of SOC on depressive symptoms among Arab students.

#### 1.2.2. Coping Strategies

Coping processes are complex responses that occur when an individual attempts to remove a source of stress or a perceived threat from his or her environment. The reaction to an event has been found to be as important as the event itself [32]. Coping strategies include cognitive or behavioral efforts to manage situations appraised as taxing or exceeding a person’s resources [3]. A common characteristic of many coping taxonomies has been the distinction between strategies that are active and oriented toward confronting the problem (i.e., active coping strategies) and strategies that entail an effort to reduce tension by avoiding dealing with the problem (i.e., avoidant coping strategies [33]). Research on the effects of coping strategies on adjustment has found that active coping strategies are more effective and that they moderate the adverse influence of negative life events on psychological functioning [34,35]. In contrast, avoidant coping strategies tend to be associated with psychological distress [33,35,36,37].

Berry [4] noted that, when acculturative stress is not managed well, it will increase and its effect will be even more negative. In addition, if such stressors become overwhelming, the immediate effects can be significantly negative and damaging, even to the point of personal crises, anxiety, and depression. When acculturative problems (stressors) arise, but are successfully managed, stress levels are similarly low and the immediate effects are positive [38].

#### 1.2.3. Coping Strategies in a Collectivistic Cultural Context

Sociocultural groups appear to generate not only consensual belief systems concerning the origin and meaning of stressors, but also beliefs concerning the most appropriate means to cope with stressors. Empirical investigations of coping strategies across cultures have yielded mixed findings. However, overall, there is significant support for the idea that individuals from collectivistic cultural contexts are more likely to use avoidant coping strategies [39,40], whereas individuals from individualistic cultural contexts are more likely to use active and problem-focused coping strategies [40,41]. Among Arab students in Israel (specifically Bedouin Arabs), levels of active coping strategies are similar to those found in Israeli Jewish society [42]. However, Arab students have also reported using more avoidant coping strategies than Jewish students [42]. This study provides support for the important role of avoidant coping strategies. Bedouin Arab students tend to be more depressed because they tend to use avoidant coping strategies more often. That is, they attempt to reduce tension by avoiding dealing with problems (i.e., behavioral disengagement, self-distraction, denial, and self-blame [42]).

### 1.3. Gender Differences in Acculturative Stress, SOC, Coping Strategies, and Depression

Gender has a variety of effects on the acculturation process. There is substantial evidence that women may be at greater risk for problems in the acculturative process than men (e.g., [43,44]). Female immigrants reported higher levels of acculturative stress than men across multiple domains including homesickness, social isolation, employment barriers, discrimination, and civic disengagement [44]. Attempts by women to take on the new roles available in the “new” society may bring them into conflict with their heritage culture, placing them at risk for acculturative stress and negative outcomes [43,45]. In addition, the majority of previous studies have reported higher SOC scores among men [20]. Gender differences have also been found in relation to coping strategies. In general, women tend to exploit social support, affective release, emotional regulation, and emotionally focused and “tend-and-befriend” strategies (which may be considered a form of active coping( [46,47]. In contrast, the coping efforts of men are directed toward “fight-or-flight” responses (i.e., gaining control over the situation and invoking disengagement responses) [47,48]. Gender differences have also been found in levels of depression. For example, women are twice as likely as men to have higher scores on self-reported depression symptom measures [49]. However, previous research among students from a subgroup belonging to the Arab minority in Israel (i.e., Bedouin Arabs) revealed no gender differences in mean levels of depressive symptoms [42,50]. It would be interesting to examine the generalizability of these findings among students from the larger Arab minority.

### 1.4. Academic-Year Differences in Acculturative Stress, SOC, Coping Strategies and Depression

Previous research suggests that students in the earlier years of their college educations are at higher risk for experiencing psychological distress and depression than students in the later years of their degree programs [51]. One possible explanation is that the period of transition from high school to college is stressful and this stress may be related to elevated rates of depression among these students [51]. Research on the effects of academic-year differences on acculturative stress among Chinese nursing students in Australia found higher levels of acculturative stress in the third year as compared to the second year. However, that study reported no significant differences in acculturative stress between first-year students and second-year students [52]. He et al. [52] compared the SOC levels among first-, second-, and third-year students and found no significant differences between the three groups. It is important to note that most the studies have focused on a single academic year [53] or have not reported comparisons of different years of study [54]. It is important to test whether students in the earlier years of college express different levels of acculturative stress and depressive symptoms. In addition, special attention should be paid to their coping resources and strategies, as compared to the resources and strategies found among students who are further along in their studies.

### 1.5. Acculturative Stress Among Arab Students in Israel

The Arab minority in Israel comprises about 21% of the entire population [55]. During the last decade (2008–2018), there was an 80% increase in the number of Arab students in academic institutions of higher learning in Israel [56]. Arab culture differs significantly from Jewish Israeli culture in terms of its emphasis on collectivistic ideals [57]. Jewish culture, being more individualistic and less authoritarian, emphasizes separation, independence, personal development, and achievement [58]. In addition, Arabs also differ from the Jewish majority in terms of language, religion, and other cultural factors [59]. This large cultural distance between the Arab minority and the Jewish majority is expected to increase the acculturative stress experienced by Arab individuals. Discrimination, prejudice, and negative stereotypes and attitudes of the host culture toward the minority group also increase acculturative stress [60]. Arabs in Israel are a largely underprivileged minority with a history of disadvantage in income, education, and employment [61]. They live in segregated residential areas [61]. Despite enjoying full citizenship status, the Arab minority is subject to various forms of discrimination that may contribute to social and economic disparities between them and the Jewish majority [62,63]. These experiences of discrimination are expected to contribute to acculturative stress among Arab students. These students doubt the readiness of the majority to welcome them and tend to feel that they are discriminated against [63]. Members of Arab society, who share more traditional and collectivist values [64], have to adjust to unfamiliar values and codes of behavior and fit in with the majority of students in a more Western-individualistic cultural milieu, which differs substantially from their native culture. To the best of our knowledge, there has been no previous research on acculturative stress within Arab society or specifically among students attending higher academic institutions within this society. Thus, the current study will address acculturative stress and its associations with depressive symptoms among female and male Arab students at different stages of their academic studies.

### 1.6. The Current Study

The main goals of the current study were to explore the associations between acculturative stress, SOC, coping strategies, and depressive symptoms among Arab students from northern and central Israel. Special attention was paid to the roles of those variables in the associations between acculturative stress and depressive symptoms. The current study also investigated gender differences in the levels and roles of SOC and the use of different coping strategies within the Arab minority in Israel. The effects of academic year (Year 1 + 2 vs. Year 3 + 4) on the levels of the study variables and the roles of SOC, active coping, and avoidant coping in the association between acculturative stress and depressive symptoms were also examined.

### 1.7. Hypotheses 

**Figure 1:**
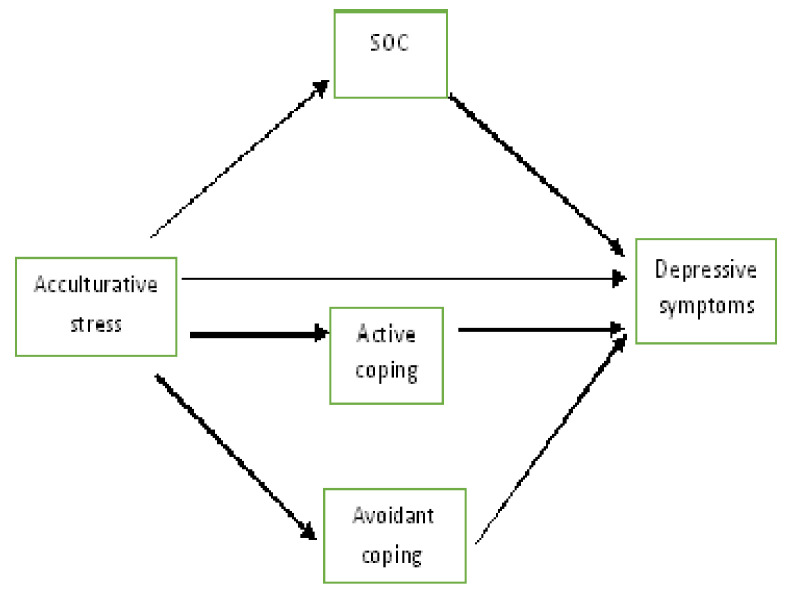
The hypothesized relationships between acculturative stress, avoidant coping, active coping, SOC, and depressive symptoms. SOC: Sense of coherence.

The following hypotheses were tested:

1. There are gender differences in acculturative stress, SOC, coping strategies, and depressive symptoms [20,42,43,44,50,65,66].
2. There are academic-year differences in acculturative stress and depressive symptoms [51,52].
3. There is a positive association between acculturative stress and depressive symptoms [13,14].
4. SOC and coping strategies mediate the relationships between acculturative stress and depressive symptoms [42,67,68]. There are gender and academic-year differences in the pathways between acculturative stress and depressive symptoms (Figure 1).

## 2. Materials and Methods

### 2.1. Participants and Procedure

We employed a cross-sectional research design. One hundred seventy individuals participated in the study, 103 female Arab students and 67 male Arab students from northern and central Israel who were studying at institutions of higher education (i.e., Ben-Gurion University of the Negev, the Hebrew University of Jerusalem, Tel Aviv University, and Haifa University).

The study was approved by the Department’s Human Subjects Ethics Committee (Approved Ethics Form No. 0015-009). Participants were approached and included in the study using convenience sampling. All participants were from the northern and central regions of Israel and currently enrolled in an institution of higher learning. Students were recruited with the cooperation of close friends of the second author of this study who study in Israeli universities and by circulating the questionnaire through the social media. Since we used social-media platforms (mainly Facebook), we could not determine the response rate. The process of data collection took three weeks. Arab students were encouraged to complete the questionnaires via a link. The first page of the linked document included an informed-consent form and only students who checked a box to indicate their agreement to participate were directed to complete the full set of questionnaires. The purpose of the research study was also presented to the participants on the first page of that document. The participants were explicitly requested to refrain from providing identifying information. After the participants finished answering the questionnaires, they were presented with a final page that included a full description of the purpose of the study, contact information for the researchers, and a list of references related to the research topic. Arabic versions of the questionnaires were used. Each participant was reimbursed with a coupon for coffee and cake.

### 2.2. Measures

#### 2.2.1. Acculturative Stress—Societal, Attitudinal, Familial, and Environmental—Revised–Short Form (SAFE-Short)

This 24-item measure is designed to assess negative stressors experienced by minority individuals. It captures both stress experienced within one’s own group and stress experienced when engaging with the mainstream culture [7]. The items are statements that describe situations that may cause stress (e.g., “It bothers me that I cannot be with my family.” and “My family members and I have different expectations about my future.”) The items are rated on a Likert scale of 1–5 (1 = not stressful; 2 = somewhat stressful; 3 = stressful; 4 = very stressful; 5 = extremely stressful). The mean score of the scale ranged from 1 to 5. This scale has been found to be reliable across different ethnic and cultural groups [69]. In the current study, the internal consistency coefficient of the scale was 0.88.

#### 2.2.2. Sense of Coherence (SOC) Questionnaire

This questionnaire consists of 13 items that measure a respondent’s perception of life as comprehensible, manageable, and meaningful [18]. The items were rated using a 7-point Likert scale that had an anchoring phrase at each end. High scores indicated a strong SOC. The scale included such items as “Doing the things you do every day is” with answers ranging from 1 (a source of pain and boredom) to 7 (a source of deep pleasure and satisfaction). The mean score of the scale ranged from 1 to 7. In this study, we used the Arabic version of the SOC questionnaire. This version has been used in several previous research projects and has been found to be reliable and suitable for use among the Bedouin Arab population [23,70,71]. In the current study, the internal consistency reliability for the scale was 0.80.

#### 2.2.3. Active and Avoidant Coping—The Coping Orientations to Problems Experienced Inventory (COPE)

The COPE-Short Form is a 28-item questionnaire used to assess different dimensions of active or avoidant coping strategies [72]. Participants rate each coping statement in terms of how frequently they use each strategy to manage stressful events, on a scale of 1 (never) to 5 (always). The subscales were aggregated to form two composite scales: active coping (14 items related to planning, instrumental support, emotional support, positive reframing, problem-solving, and humor) and avoidant coping (14 items related to self-blame, behavioral disengagement, self-distraction, substance use, and denial). Coping statements include items such as “I spent more time alone”; “I blamed myself”; “I tried to forget the whole thing”; “I’ve been getting emotional support from others”; and “I’ve been getting comfort from someone.” The mean scores of the active-coping and avoidant-coping strategy subscales ranged from 1 to 5. In the current study, the Arabic version of the instrument [73] was used. The internal consistency coefficients of the active and avoidant coping scales of the instrument were 0.83 and 0.71, respectively.

#### 2.2.4. Depressive Symptoms—The Center for Epidemiological Studies Depression Scale (CES-D)

The CES-D Scale is a 20-item inventory of symptoms of depression [74]. Respondents report how frequently symptoms have been experienced during the past month, using a 4-point Likert scale that ranges from 0 (rarely or none of the time; less than once a day) to 3 (most or all of the time; 5–7 days a week). Items include: “I was bothered by things that usually don’t bother me” and “I felt depressed.” The total score of the scale ranged from 0 to 60. Score above the stricter diagnostic cut-off point of 23 indicate severe levels of depressive symptoms [75]. In our previous studies, the Cronbach’s internal consistency alpha coefficients for the Arabic version were around 0.90 [50]. In the current study, the internal consistency coefficient of the scale was 0.93.

#### 2.2.5. Demographics

Participants were asked to report their gender, age, marital status, institutional affiliation and academic year, parents’ levels of education, and household income. The variable academic year was recoded to new variable; students in their first and second academic year were included in the group Year 1 + 2 and students in their third and fourth academic year were included in the group Year 3 + 4.

### 2.3. Data Analysis

The collected data were analyzed using SPSS (IBM SPSS Statistics 26.0, Chicago, IL, USA) and structural equation modeling (SEM), which was carried out using SPSS AMOS 26 software [76]. Three sets of analyses were conducted. First, to test the gender and academic-year differences, we conducted two independent-sample *t*-tests with five dependent variables: acculturative stress, SOC, active coping, avoidant coping, and depressive symptoms. The second hypothesis was tested using Pearson correlations, which involved acculturative stress, depression, SOC, and coping strategies. We used SEM to test the direct and direct effects of acculturative stress on depressive symptoms via SOC and coping strategies among female and male Arab students, as well as among Year 1 + 2 and Year 3 + 4 Arab students.

## 3. Results

### 3.1. The Study Population

One hundred seventy individuals participated in the study, 103 female Arab students and 67 male Arab students. The participants’ had a mean age of 21.88 years (*SD* = 2.54). The majority of the students were single and the children of parents who had 12 or fewer years of education (82.4%). A complete description of the demographic characteristics of the study population is presented in Table 1.

### 3.2. Gender Differences in Levels of Acculturative Stress, SOC, Coping Strategies, and Depression

Our first hypothesis was that gender differences would be related to levels of acculturative stress, coping strategies, and depression. To test that hypothesis, we conducted independent-sample *t*-tests with five dependent variables (i.e., acculturative stress, SOC, active coping, avoidant coping, and depressive symptoms). This analysis revealed significant gender differences in the use of active and avoidant coping strategies, as well as depressive symptoms. In addition, non-significant gender differences were found in the levels of acculturative stress and SOC.

As shown in Table 2, female Arab students reported higher levels of avoidant coping, active coping, and depressive symptoms, as compared to male students. Concerning depressive symptoms, forty-five (44%) of the female participants had mean CES-D scores that exceeded the stricter diagnostic cut-off point of 23. In contrast, 22 (33%) of the male participants had CES-D scores above the stricter diagnostic cut-off point.

### 3.3. Academic-Year Differences in Levels of Acculturative Stress, SOC, Coping Strategies, and Depression

Our second hypothesis was that academic year differences would be related to levels of acculturative stress and depression. To test that hypothesis, we conducted independent-sample *t*-tests with five dependent variables (i.e., acculturative stress, SOC, active coping, avoidant coping, and depressive symptoms). This analysis revealed significant academic year differences only in depressive symptoms. In addition, non-significant gender differences were found in the levels of acculturative stress. As shown in Table 3, Year 1 + 2 Arab students reported higher levels of depressive symptoms as compared to Year 3 + 4 students.

### 3.4. Relationships Between the Study Variables Among the Students

We computed Pearson’s correlations between the study variables. As shown in Table 4, a positive association was found between acculturative stress and depressive symptoms. In addition, avoidant coping was found to be positively associated with acculturative stress. In other words, higher levels of acculturative stress were related to both greater use of avoidant coping strategies and more depressive symptoms. In addition, negative associations were found between acculturative stress and SOC and active coping. Higher levels of acculturative stress were related to lower levels of SOC and less use of active coping strategies among Arab students.

### 3.5. Direct and Indirect Relationships between Acculturative Stress and Depression among Male and Female Arab Students

Multiple-group SEM analysis was performed with SPSS AMOS software [76], using the maximum-likelihood estimation to test how well the data fit the hypothesized model. AMOS generates a variety of indices for evaluating fit; models with chi-square/degrees of freedom ratios of less than two considered acceptable. We also employed the non-normed fit index (NNFI) [77], the Tucker–Lewis index (TLI), the comparative fit index (CFI) [78], and the root mean square error of approximation (RMSEA). Index values between 0.00 and 0.08 are generally deemed acceptable [79]. The fit indices of the hypothesized model were as follows: CFI = 0.99, NNFI = 0.91, RMSEA = 0.07, CMIN/*df* = 1.83, and *p* < 0.05. Thus, the hypothesized model fit the data well.

**Figure 2:**
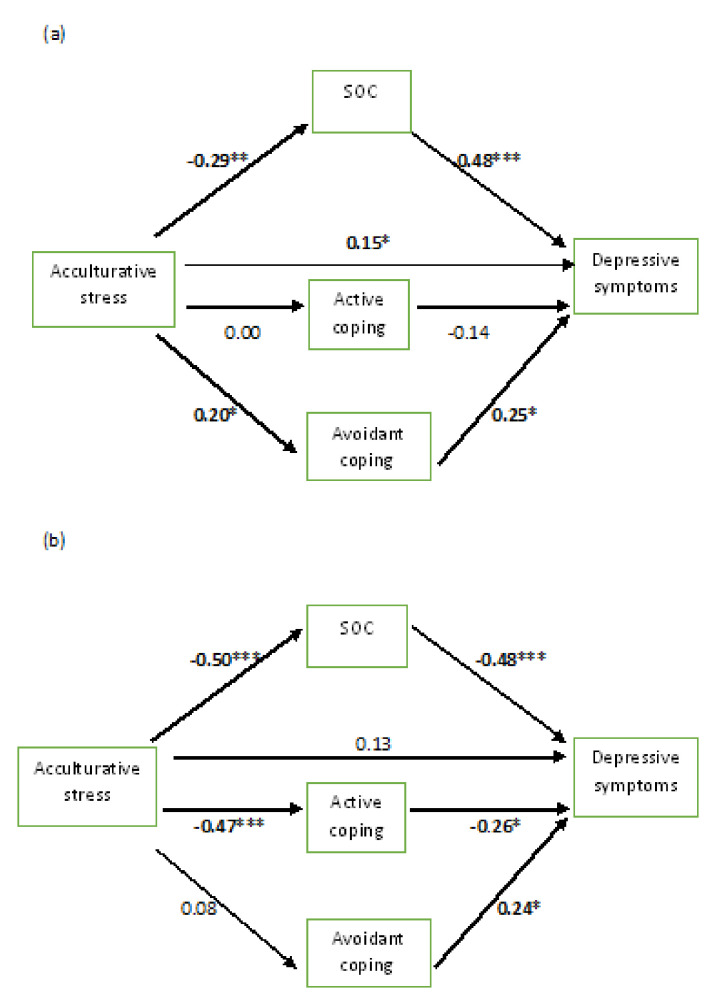
Direct and indirect effects of acculturative stress on depressive symptoms through SOC, avoidant coping, and active coping among: (**a**) female Arab students; and (**b**) male Arab students. *Note.* All of the coefficients in the figures are standardized. * *p* < 0.05; ** *p* < 0.01; *** *p* < 0.001; SOC, sense of coherence.

The full models explained 48% and 56% of the variance of depressive symptoms among female and male students, respectively. All of the coefficients reported in the text and in Figure 2 are standardized. As shown in Figure 2, among the female participants, acculturative stress had a significant direct effect (β = 0.15) and an indirect effect (β = 0.20) on depressive symptoms via SOC and avoidant coping. However, among the male participants, we observed a stronger indirect effect of acculturative stress on depression (β = 0.40) via SOC and active coping. Among the men, we did not find any significant direct effect of acculturative stress on depressive symptoms (β = 0.13).

Among the women, acculturative stress had significant direct effects on SOC (β = −0.29) and avoidant coping (β = 0.20). Moreover, we observed significant direct effects of SOC (β = −0.48) and avoidant coping (β = 0.25) on depressive symptoms. In comparison, among the men, acculturative stress had strong direct effects on SOC (β = −0.50) and active coping (β = −0.47). We also observed significant direct effects of SOC (β = −0.48) and active coping (β = −0.26) on depressive symptoms among the men. The results from this analysis underscore the significance of the indirect effect of acculturative stress on depressive symptoms through SOC and coping strategies among female and male Arab students. However, our results support the direct effect of acculturative stress on depression among female students, but not among male students.

### 3.6. Direct and Indirect Relationships between Acculturative Stress and Depression among Year 1 + 2 and Year 3 + 4 Arab Students

Multiple-group SEM analysis was performed with SPSS AMOS software to compare the effects of the different variables on depressive symptoms in the two groups of Year 1 + 2 and Year 3 + 4 Arab Students. The fit indices of the hypothesized model were as follows: CFI = 0.98, NNFI = 0.93, RMSEA = 0.08, CMIN/*df* = 1.94, and *p* < 0.05. Thus, the hypothesized model fit the data well.

**Figure 3:**
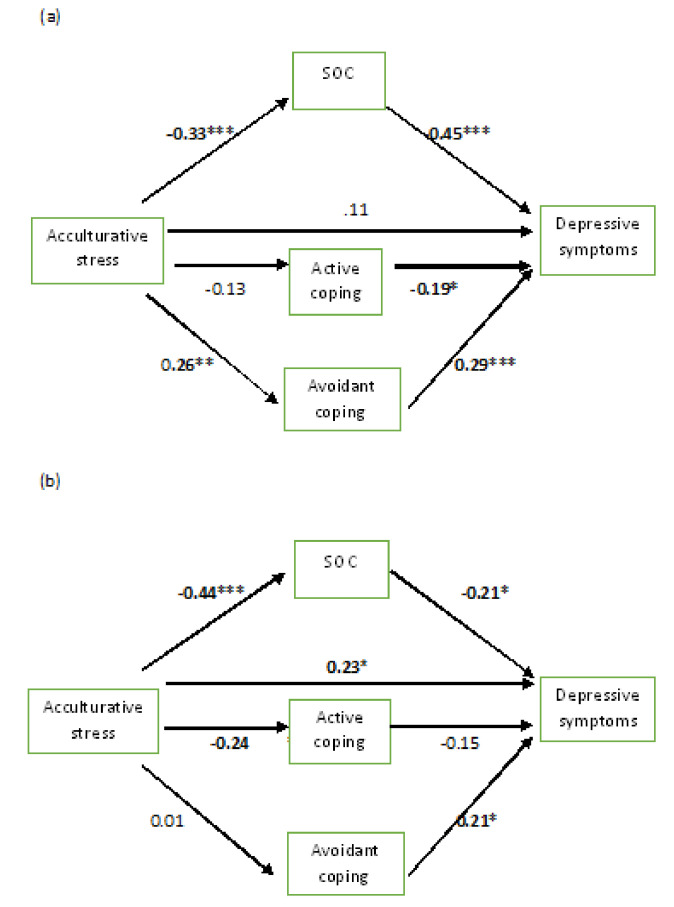
Direct and indirect effects of acculturative stress on depressive symptoms through SOC, avoidant coping, and active coping among: (**a**) Year 1 + 2 Arab students; and (**b**) Year 3 + 4 Arab students. *Note*. All of the coefficients in the figures are standardized. * *p* < 0.05; ** *p* < 0.01; *** *p* < 0.001; SOC, sense of coherence.

The full models explained 45% and 53% of the variance of depressive symptoms among Year 1 + 2 students and Year 3 + 4 students, respectively. All of the coefficients reported in the text and in Figure 2 are standardized. As shown in Figure 3, among the Year 1 + 2 participants, acculturative stress had an indirect effect (β = 0.27) on depressive symptoms via SOC and avoidant coping. However, among the Year 3 + 4 participants, we observed a direct effect of acculturative stress on depression (β = 0.23) and an indirect effect of acculturative stress on depression (β = 0.22) via SOC. Among the Year 1 + 2 students, we did not find any significant direct effect of acculturative stress on depressive symptoms (β = 0.11).

Among the Year 1 + 2 students, acculturative stress had significant direct effects on SOC (β = −0.33) and avoidant coping (β = 0.26). Moreover, we observed significant direct effects of SOC (β = −0.46), active coping (β = −0.19), and avoidant coping (β = 0.29) on depressive symptoms among those students. In comparison, among the Year 3 + 4 students, acculturative stress had strong direct effects on SOC (β = −0.44) and active coping (β = −0.24). We also observed significant direct effects of SOC (β = −0.42) and avoidant coping (β = 0.21) on depressive symptoms among the Year 3 + 4 students.

The results of these analyses underscore the significance of the indirect effect of acculturative stress on depressive symptoms, through SOC and avoidant-coping strategies, among Year 1 + 2 Arab students. However, our results support the direct effect and the indirect effect of acculturative stress on depressive symptoms through SOC only among Year 3 + 4 Arab students.

An examination of the total (direct and indirect) effects of acculturative stress revealed a meaningful picture. Among the female participants, acculturative stress affected depressive symptoms directly and indirectly through SOC and avoidant coping. However, among Arab male participants, acculturative stress affected depressive symptoms only indirectly, through SOC and active coping. Moreover, among Year 1 + 2 students, acculturative stress was related to depressive symptoms indirectly via SOC and avoidant coping. However, among the Year 3 + 4 students, acculturative stress was related to depressive symptoms both directly and indirectly via SOC.

## 4. Discussion

The main aim of this study was to examine the association between acculturative stress and depressive symptoms. We were interested in exploring the roles of SOC and avoidant and active coping strategies in this relationship among male and female Arab college students from northern and central Israel who are at different stages of their studies in Israeli institutions of higher education. Important findings emerged from this study. Gender differences were found in both active and avoidant coping, as well as in depressive symptoms. Academic-year differences were found in SOC and depressive symptoms. In addition, higher levels of acculturative stress were found to be related to lower levels of SOC, less use of active coping, more use of avoidant coping, and higher levels of depressive symptoms. Among the female Arab students, acculturative stress affected depressive symptoms both directly and indirectly through SOC and avoidant coping. However, among Arab males, acculturative stress only affected depressive symptoms indirectly, through SOC and active coping. Among Year 1 + 2 students, acculturative stress affected depressive symptoms indirectly via SOC and avoidant coping. However, among the Year 3 + 4 students, acculturative stress affected depressive symptoms both directly and indirectly via SOC.

### 4.1. Gender Differences in Coping Strategies

Gender differences were found in both active and avoidant coping, with females scoring higher in both active and avoidant coping than their male counterparts. This is consistent with previous literature that has noted that coping mechanisms may be gender-specific [80] and that females tend to face higher levels of stress, which are associated with the use of more coping resources [66]. Abu-Kaf and Braun-Lewensohn [42] confirmed that female Bedouin Arab students report greater use of both active coping and avoidant coping, as compared to male Bedouin Arab students. Bedouin Arab females reported using a variety of coping strategies as they confront problems/stressors (i.e., social support, emotional regulation, affective release, and emotion-focused strategies). They also attempt to reduce tension by avoiding dealing with problems (i.e., behavioral disengagement, self-distraction, denial, and self-blame). This finding may be related to the understanding that females suffer disproportionately from life stressors. Understanding that difference is essential for the discussion of gender differences in personal methods of coping and efforts to overcome stressful situations [3]. In other words, the greater number of life stressors that Arab females experience may be associated with the use of more coping mechanisms, whether avoidant or active [66].

### 4.2. Gender Differences in Depressive Symptoms

In terms of depressive symptoms, the current study revealed a higher monthly prevalence rate, as well as higher scores (mean) among female Arab students, as compared to male Arab students. These finding provide more support for previous research, which found gender differences in depression. For example, women are twice as likely as men to have higher scores on self-reported measures of depressive symptoms [49]. It is important to mention that mean depression scores and the monthly prevalence rate among male Arab students were higher than those usually observed among male and female student samples in Israel. Previous research found that the reported rates of severe levels of depressive symptoms range from 12.5% to 16.7% among male Jewish students and range from 15.1% to 17% among Jewish female students [50]. In the current study, about one-third of the Arab male students scored above the stricter diagnostic cut-off point of 23, indicating severe levels of depressive symptoms. Mental-health problems have been found to be more prevalent among Arab students, particularly among female Arab students [42,81]. Depressive symptoms among students have been found to affect learning and memory processes, leading to lower levels of academic achievement [82], poor attendance, failure to complete the academic degree [83], dropping out of the academic institution [84], and even suicidal ideation [85].

### 4.3. Academic-Year Differences in Depressive Symptoms

Our results revealed differences in depressive symptoms among Year 1 + 2 students as compared to Year 3 + 4 students. Year 1 + 2 students reported higher levels of depressive symptoms than Year 3 + 4 students. This finding supports previous research which addressed the vulnerability of students in the earlier years of their college educations to psychological distress, specifically depressive symptoms [51]. Previous studies have demonstrated that the period of transition from high school to college is very stressful for many students, in general, and for student from cultural-ethnic minority groups, in particular [50,62].

### 4.4. Academic-Year Differences in SOC

Another interesting finding is related to the higher levels of SOC observed among Year 3 + 4 students, as compared to Year 1 + 2 students. This finding does not support the findings of a previous study that found no academic-year differences in SOC among Chinese nursing students in Australia. Our finding may be explained by the fact that more advanced Arab students have more experience, greater knowledge about the academic environment, and larger social networks on campus than students who are in the earlier stages of their education [86]. This may affect their perception of college life and the academic environment as structured and predictable, as well as increase their capacity to assess and use available resources to face and cope with stressful situations [21].

### 4.5. Associations Between Acculturative Stress and Depressive Symptoms

Our findings revealed that acculturative stress is positively related to depressive symptoms. This finding supports previous research that has found acculturative stress to be associated with mental-health problems, including anxiety and depression [2,10,11,12]. Among college students (international students and students from a cultural-ethnic minority), acculturative stress has been associated with a number of psychological challenges, including depression [13,14]. This finding may be related to the associations that have been found between acculturative stress and feelings of alienation, being discriminated against, and feeling that one does not belong, which may contribute to feelings of disconnection and depression [87].

### 4.6. The Direct and Indirect Effects of SOC and Coping Strategies on the Association Between Acculturative Stress and Depressive Symptoms

Among both males and females, acculturative stress had an indirect effect on depressive symptoms via SOC. This confirms the result of no gender bias in the levels of SOC in this study. Many cross-sectional studies have reported a significant inverse correlation between SOC and depression. According to Sairenchi, Haruyama, Ishikawa, Wada, Kimura, and Muto [88], SOC can predict the onset of depression in Japanese workers. Workers with low levels of SOC might suffer from depression more than workers with high SOC; no gender differences were found in these associations. Therefore, examining the levels of SOC among students of both genders may be useful for identifying Arab students at high risk of future depressive symptoms.

Among female Arab students, the effect of acculturative stress was amplified through the indirect effects of avoidant coping. Among the male students, the effect of acculturative stress was amplified through the indirect effects of active coping. Our findings underscore previous claims that passive/avoidant coping is strongly related to general psychological distress and depression [42] and that active coping is inversely related to depression [89].

The fact that cultures vary in the degree to which gender roles are emphasized could contribute to this difference. Williams and Best [90] confirmed that men and women in traditional-collectivistic cultures (i.e., Arab culture, in this case) tend to emphasize gender-role differences, whereas those in more Western-individualistic cultures tend to minimize them. Presumably, such values could affect the development of gender differences, in general, and the direct and indirect effects of acculturative stress on depressive symptoms, in particular. Thus, Arab males, who are seen in their patriarchal society as the main providers and guardians of their families [57], may be driven to use active coping strategies as they confront challenges. Among Arab males, acculturative stress may lead to more depressive symptoms by affecting (i.e., decreasing) their use of active coping strategies. Arab women are expected to adopt more avoidant/passive coping strategies, in order to fit in their social roles and fulfill their obligations in more traditional cultural contexts [57]. Among these women, acculturative stress may lead to more depressive symptoms by affecting (i.e., increasing) their use of avoidant coping strategies.

Among both Year 1 + 2 and Year 3 + 4 Arab students, acculturative stress had an indirect effect on depressive symptoms via SOC. This finding confirms the findings of previous studies conducted among Bedouin Arabs, which found that, over time, SOC becomes a strong predictor of stress reactions [24]. Among Year 1 + 2 Arab students, acculturative stress had an indirect effect on depressive symptoms via the use of avoidant-coping strategies. Similar to what was observed for female Arab students, the role of avoidant coping in the association between acculturative stress and depressive symptoms underscores previous findings that indicated that passive/avoidant coping is strongly related to general psychological distress and depression [42].

The present study suggests that Arab individuals of both genders and across different stages in their academic studies who face relatively high levels of acculturative stress tend to exhibit higher levels of depressive symptoms, but that the effect of acculturative stress on depression develops via similar, as well as different pathways among female and male, and among more junior (Year 1 + 2) and more advanced (Year 3 + 4) Arab students. The female Arab students tend to be more depressed as a direct result of their higher levels of acculturative stress, which arise from the need to learn new and sometimes confusing cultural rules and expectations, deal with experiences of prejudice and discrimination, and manage the overarching conflict between maintaining elements of their old culture while incorporating elements of the new. These depression levels are also an indirect result of their lower levels of SOC, as well as their stronger tendency to use avoidant coping strategies (i.e., to attempt to reduce tension by avoiding dealing with problems through behavioral disengagement, self-distraction, denial, and self-blame). In contrast, male Arab students experiencing high levels of acculturative stress tend to be more depressed as an indirect result of their lower levels of SOC and their limited use of active coping strategies (i.e., planning, instrumental support, emotional support, positive reframing, problem-solving, and humor). Year 1 + 2 students experiencing high levels of acculturative stress tend to be more depressed as an indirect result of their lower levels of SOC and their increased use of avoidant coping strategies (like the female students, they tend to increase their use of behavioral disengagement, self-distraction, denial, and self-blame). However, Year 3 + 4 Arab students tend to be more depressed as a direct result of their higher levels of acculturative stress and also as an indirect result of their lower levels of SOC.

The current study contributes to the understanding of gender and academic-year differences in coping resources and the use of different strategies to deal with acculturative stressors in the context of higher education. This research suggests that female and male students, as well as students at different stages of their degree programs, from a more traditional, Eastern-collectivistic cultural background who exhibit high levels of acculturative stress tend to have different and distinct pathways to emotional distress. However, because the research design is cross-sectional, we cannot completely exclude the possibility that depression may lead to greater acculturative stress and lower SOC, as well as lowered motivation to deal actively with stressors and high levels of avoidance and withdrawal.

### 4.7. Limitations and Directions for Future Research

Although the fact that this work may be the first study of acculturative stress and depressive symptoms among Arab students in Israel underscores its value, it also has several limitations that should be taken into account when considering the research findings. There are also areas that warrant further attention in future research. First, the study design was cross-sectional and, therefore, we cannot make any claims of causality. In addition, we did not evaluate past depressive experiences among those students, an important variable that may affect the prospective levels of acculturative stress and depressive symptoms during their academic studies. Thus, future research studies should employ a longitudinal design and aim to test prior levels of depressive symptoms, as well as the prospective relationship between acculturative stress and depressive symptoms in the academic setting. Second, our data were based on self-report measures, which have particular limitations, such as restrictive rating scales, and can be limited by the introspective ability of participants, inaccurate interpretations of particular questions, and biased responses. In future research, the use of other methods of data collection (especially diaries and interviews) would be beneficial and important for the evaluation of the validity of the obtained findings. Third, the study involved participants from only four of the many institutions of higher education in Israel: Ben-Gurion University of the Negev, Tel Aviv University, The Hebrew University of Jerusalem, and Haifa University. Therefore, future research should include students from wider range of Israeli academic institutions and the generalizability of the current finding should be evaluated. Another avenue for future research might be to extend the present model by examining cultural factors such as self-construal, collectivism level, and somatization, which may be important indicators of psychological distress in Arab cultural contexts [50,91,92].

## 5. Conclusions

The importance of the current study lies in its examination of the roles of coping resources and coping strategies in the association between acculturative stress and depressive symptoms among female and male, as well as more junior and more advanced, Arab college students. The present study highlights the importance of SOC, as well as active coping and avoidant coping, in the relationship between acculturative stress and depressive symptoms. Gender and academic-year similarity was found with regard to the role of SOC, whereas differences were found in the direct effects and the roles of active and avoidant coping. Among the male students, active coping played a significant role in the association between acculturative stress and depressive symptoms. In contrast, among female and Year 1 + 2 students, avoidant coping played a significant role. This knowledge is expected to help the employees of academic departments understand the different aspects of the coping and distress that characterize Arab students. Male and female students and students at different stages of their degree programs may need different interventions to help them adjust to academic life, the acculturation process, and the stresses of this process. Psychological counseling and guidance programs should be tailored to the specific needs of Arab students, with special attention given to the roles of gender, academic year, coping resources, and coping strategies.

## Figures and Tables

**Table 1:** Frequencies and percentages of demographic variables.

Demographic Variable	Frequency	Percentage (%)
Gender		
Women	103	69.5
Men	67	30.5
Marital status		
Single	140	82.4
Engaged	15	8.8
Married	14	8.2
Divorced	1	0.6
Widow/er		
Academic year		
First	48	28.2
Second	50	29.4
Third	44	25.9
Fourth	28	16.5
Household income		
Much more than average	6	3.5
More than average	38	22.4
Similar to average	55	32.4
Less than average	39	22.9
Much less than average	32	18.8

**Table 2:** Gender differences in acculturative stress, SOC, active coping, avoidant coping, and depressive symptoms.

Variable	Male *n* = 67		Female *n* = 103		*t-*Value	Hedges’ *g*
	*M*	*SD*	*M*	*SD*		
Acculturative stress (0–5)	1.80	0.86	1.84	0.78	−0.27	0.04
SOC (1–7)	4.28	0.90	4.08	0.97	1.35	0.21
Coping strategies						
Active coping (1–5)	2.40	0.42	2.60	0.47	−2.88 *	0.45
Avoidant coping (1–5)	2.0	0.41	2.20	0.45	−3.08 **	0.48
Depressive symptoms (0–60)	18.64	10.86	23.47	13.41	−2.29 *	0.31

* *p* < 0.05; ** *p* < 0.01; SOC, sense of coherence.

**Table 3:** Academic-year differences in acculturative stress, SOC, active coping, avoidant coping, and depressive symptoms.

Variable	Year 1 + 2 Arab Students *n* = 98		Year 3 + 4 Arab Students *n* = 72		*t-*Value	Hedges’ *g*
	*M*	*SD*	*M*	*SD*		
Acculturative stress (0–5)	1.81	0.81	1.83	0.82	−0.15	0.02
SOC (1–7)	4.02	0.93	4.34	0.93	−2.28 *	0.35
Coping strategies						
Active coping (1–5)	2.48	0.48	2.52	0.44	−0.45	0.07
Avoidant coping (1–5)	2.16	0.49	2.03	0.37	1.91	0.30
Depressive symptoms (0–60)	23.42	13.05	19.04	11.74	2.25 *	0.35

* *p* < 0.05; SOC, sense of coherence.

**Table 4:** Pearson’s correlations between the study variables.

Variables	1	2	3	4
1. Acculturative stress				
2. SOC	−0.37 ***			
3. Avoidant coping	0.16 *	−0.41 ***		
4. Active coping	−0.17 *	0.20 *	0.23 **	
5. Depressive symptoms	0.40 ***	−0.69 ***	0.44 ***	−0.24 **

* *p* < 0.05; ** *p* < 0.01; *** *p* < 0.001; SOC, sense of coherence.
